# Signal Transducer and Activator of Transcription 3 (STAT3) Variant p.K709N Causes Hyper‐IgE Syndrome Likely by Impaired STAT3‐Dimer Formation

**DOI:** 10.1002/eji.70015

**Published:** 2025-07-28

**Authors:** Beate Hagl, Benedikt D. Spielberger, Betina Neumann, Simon J. Pelham, Dharmendra Pandey, Andreas Schlundt, Camille Barro, Anica Lechner, Christine Wolf, Elissa K. Deenick, Michael Sattler, Stuart G. Tangye, Simon Rothenfusser, Ellen D. Renner

**Affiliations:** ^1^ Translational Immunology, Faculty of Medicine University of Augsburg Augsburg Germany; ^2^ Translational Immunology in Environmental Medicine, School of Medicine and Health Technical University of Munich Munich Germany; ^3^ Institute of Lung Health and Immunity (LHI), Helmholtz Munich, Comprehensive Pneumology Center (CPC‐M), German Center of Lung Research (DZL) Munich Germany; ^4^ Department of Pediatrics, TUM University Hospital, School of Medicine and Health Technical University of Munich Munich Germany; ^5^ Department of Pediatrics, Dr. Von Hauner Children's Hospital LMU University Hospital, LMU Munich Munich Germany; ^6^ Garvan Institute of Medical Research Sydney Australia; ^7^ Faculty of Medicine and Health UNSW Sydney Sydney Australia; ^8^ Division of Clinical Pharmacology LMU University Hospital, LMU Munich Munich Germany; ^9^ TUM School of Natural Sciences, Bavarian NMR Center and Department of Bioscience Technical University of Munich Garching Germany; ^10^ Institute of Structural Biology, Molecular Targets and Therapeutics Center Helmholtz Munich Neuherberg Germany; ^11^ Institute for Biochemistry University of Greifswald Greifswald Germany; ^12^ Immunodeficiency clinic, Division of Infectious Diseases, Department of Medicine IV LMU University Hospital, LMU Munich Munich Germany

**Keywords:** dimerization, HIES, Hyper‐IgE syndrome, STAT3, variant classification

## Abstract

STAT3‐hyper‐IgE syndrome (STAT3‐HIES) is an inborn error of immunity caused by heterozygous dominant‐negative mutations in the signal transducer and activator of transcription 3 (STAT3). In this study, we evaluate the functional relevance of a previously undescribed heterozygous *STAT3* variant in a patient with clinical findings of STAT3‐HIES. Flow cytometry, quantitative real‐time PCR, pull‐down assays, native PAGE, DNA‐binding ELISA, and 3D‐structural data analysis were performed. Genetic analysis identified the heterozygous *STAT3* variant NM_139276.2:c.2127G>C (NP_644805.1:p.(K709N); short: p.K709N) in a patient with a clinical and laboratory phenotype characteristic of STAT3‐HIES, including early onset severe eczema, chronic lung disease, eosinophilia, and elevated serum IgE levels. While STAT3 p.K709N did not significantly affect STAT3 phosphorylation, STAT3 target gene expression was impaired in patient cells. Expression of STAT3 p.K709N and wild‐type STAT3 in STAT3‐deficient cells indicated a dominant‐negative effect by the mutation. Analysis of 3D‐structural data and modeling suggested a central role of the affected amino acid K709 in stabilizing a C‐terminal loop in STAT3 essential for dimer formation. Consequently, p.K709N resulted in diminished STAT3 dimerization and reduced DNA binding in patient cells. Functional analyses verified STAT3 p.K709N to cause STAT3‐HIES and suggest that STAT3 p.K709N impairs STAT3 dimer formation.

## Graphical abstract

In a patient with the classical clinical phenotype of STAT3‐hyper‐IgE syndrome, we identified the previously unreported STAT3 variant p.K709N to be disease‐causing. Molecular analyses revealed that STAT3 p.K709N exerts a dominant‐negative effect, leading to impaired STAT3 dimerization and reduced STAT3 target gene expression. *Source*: Created in BioRender. Hagl B. (2025) https://BioRender.com/80tycxp.

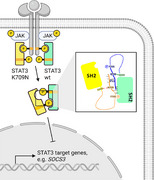

**Abbreviations**
CTT: C‐terminal tail
HIES: hyper‐IgE syndrome
IgE: immunoglobulin E
IL: interleukin
NGS: next‐generation sequencing
STAT3: signal transducer and activator of transcription 3
WES: whole‐exome sequencing
wt: wild type

## 1 Introduction

The increased availability of targeted next‐generation sequencing (NGS) and whole‐exome sequencing (WES) has revolutionized the diagnostic approach to various inborn diseases, including inborn errors of immunity (IEI) [1, 2]. Prior to using targeted NGS and WES for routine molecular diagnostics, a variety of functional diagnostic tests were performed to narrow down candidate genes. Next, a time‐consuming process of sequencing candidate genes exon by exon using conventional Sanger sequencing started. Targeted NGS and WES significantly accelerated genetic diagnosis, especially of known disease‐causing variants [2]. During NGS and WES analysis, unknown variants are evaluated by impact prediction algorithms. Although these algorithms are continuously improving, for variants with unclear functional consequences, a thorough molecular work‐up is still indispensable, as real‐life data are crucial to test and train prediction algorithms [3, 4].

Germline heterozygous dominant‐negative mutations in signal transducer and activator of transcription 3 (*STAT3*) cause autosomal‐dominant hyper‐IgE syndrome (STAT3‐HIES) [5, 6, 7]. STAT3‐HIES is an inborn error of immunity presenting with a variety of clinical and laboratory findings, including early‐onset eczema, elevated serum immunoglobulin E (IgE), and eosinophilia [5, 6, 7, 8, 9, 10]. Recurrent sinopulmonary infections lead to persistent lung tissue changes such as pneumatocele formation, which facilitate pulmonary exacerbations and secondary infections [11, 12]. Characteristic facies as well as skeletal and connective tissue anomalies like retained primary teeth, hyperflexible joints, scoliosis, and minimal trauma fractures complete the clinical presentation [5, 6, 8, 9, 10, 13].

STAT3 signaling regulates various cellular processes, including cell proliferation, differentiation, and inflammation [14]. STAT3 signaling is activated by cytokines such as interleukin (IL)‐6, IL‐10, and IL‐21 [8, 15, 16]. Upon activation, STAT3 is phosphorylated, dimerizes, and translocates to the nucleus, where STAT3 acts as a transcription factor. In STAT3‐HIES patients, the function of STAT3 is impaired due to the dominant‐negative effect of the patient's heterozygous mutation. As a consequence, STAT3‐HIES patients show reduced memory B and Th17 cell counts [8, 9, 10, 17, 18, 19].

Here we provide the functional analysis of a *STAT3* variant, which, upon detection, had been predicted to be benign or of low impact by most variant impact prediction algorithms, although the patient presented with a clinical phenotype of STAT3‐HIES. Furthermore, we show how incorporating three‐dimensional structural data and modeling suggested a molecular basis for the underlying functional defect.

## 2 Methods

See Supporting information.

## 3 Results and Discussion

### 3.1 Clinical Presentation and Genetic Analysis

**FIGURE 1:**
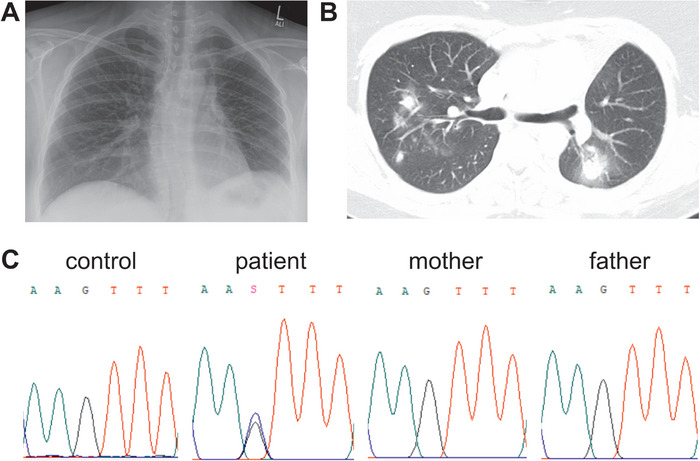
Clinical presentation and genetic analysis of the reported patient. (A) Chest X‐ray of the reported patient at 27 years of age showing an elevated left diaphragm, leftward displacement of the heart and mediastinum after left upper lung lobectomy, and scoliosis. (B) Chest CT‐scan of the patient at age 28 showing bronchial thickening as a sign of recurring infections. (C) Chromatograms show the *STAT3* wild‐type sequence in a healthy control and the patient's parents, and a double peak at NM_139276.2:c.2127G>C in the patient's genomic DNA.

We report a 34‐year‐old female patient with a characteristic phenotype of STAT3‐HIES. The patient presented with classical findings of STAT3‐HIES, including early onset severe eczema, elevated IgE serum levels, and eosinophilia. Skeletal features included persistence of primary teeth, characteristic facies, hyperflexible joints, minimum trauma fractures of the ribs, and thoracic scoliosis (Figure 1A). She had recurrent sinusitis, suffered from several episodes of pneumonia, and took prophylactic antibiotic and antimycotic treatment with trimethoprim/sulfamethoxazole and itraconazole. A severe episode of pneumonia led to lung abscess formation, followed by surgical removal of the upper lobe of the left lung at 26 years of age. Chest computed tomography scans at age 28 revealed signs of bronchial thickening from recurrent pulmonary infections (Figure 1B), an elevated left hemidiaphragm, and leftward displacement of the heart and mediastinum due to upper lobe resection. Additionally, pseudarthrosis formation was noted, resulting from recurrent rib fractures. The immunological work‐up of the patient showed reduced Th17 cells (0.08% of CD4+ cells; normal values >0.2% of CD4+ cells), elevated eosinophils (9% of leukocytes; normal range <5% of leukocytes) elevated serum IgE (range: 10124 to 20989 IU/mL; normal value <100 IU/mL), while there was a normal overall T‐ and B‐cell development including subsets, when compared with age matched references. The NIH HIES‐score, which adds characteristic findings and points toward HIES when >40 points are reached, was 66 points at maximum disease severity [20].

Sequencing of *STAT3* revealed a private heterozygous missense alteration in *STAT3* at NM_139276.2:c.2127G>C, leading to an amino acid substitution of lysine to asparagine (NP_644805.1:p.(K709N); short: p.K709N) (Figure 1C). This variant was not identified in the patient's parents, who exhibited no clinical signs of STAT3‐HIES. Furthermore, whole‐exome sequencing of the patient revealed no additional variants of interest, particularly in genes associated with HIES or other inborn errors of immunity. The STAT3 p.K709N variant was neither listed in the gnomAD nor the ClinVar database [21, 22]. Upon detection of the variant, a benign effect or low impact of the p.K709N variant on STAT3 function was predicted by most variant impact prediction algorithms. During the publication process, additional algorithms became available, and, currently, four of eight prediction tools classify the STAT3 p.K709N variant as benign or of low impact (Supporting Information Table).

Given the patient's characteristic clinical phenotype, the absence of other gene variants associated with HIES or other inborn errors of immunity, and the recognized variability and limited reliability of in silico predictions for STAT3 function [23], we conducted functional assays to determine whether the heterozygous STAT3 p.K709N variant is disease‐causing.

### 3.2 STAT3 p.K709N Does Not Affect STAT3 Phosphorylation

**FIGURE 2:**
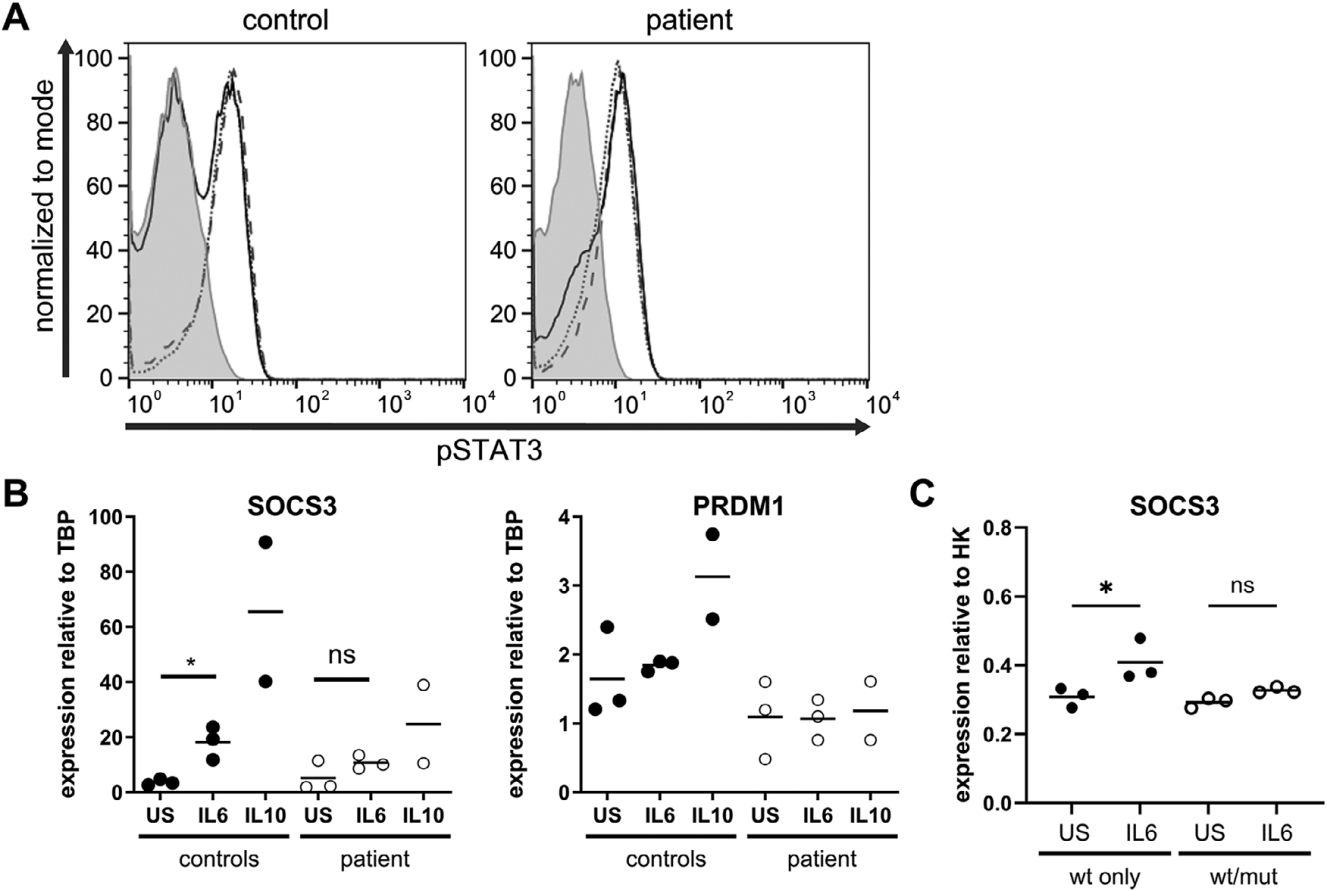
STAT3 phosphorylation and target gene expression. (A) Flow cytometric analysis of STAT3 phosphorylation (Y705) in PBMCs of a healthy individual and the patient stimulated with IL6 (solid line), IL10 (dotted line), or IL21 (dashed line) for 20 min or left untreated (filled gray area). An exemplary histogram indicating the Alexa Flour 647‐pSTAT3 signal of gated lymphocytes of *n* = 3 or 4 biological replicates is shown. Data are normalized to the mode, showing values as a percentage of the maximum count. (B) Expression of the STAT3 target genes *SOCS3* and *PRDM1* relative to the housekeeping gene *TBP* in patient or healthy control PBMCs was analyzed by qPCR after 1 h stimulation with IL6 or IL10. (C) Expression of *SOCS3* relative to the housekeeping genes (HK) *TBP* and *b‐Actin* in PC‐3 cells transfected with either wt or equal amounts of wt and p.K709N mut STAT3 plasmids. 30 h after transfection, cells were stimulated with IL6 for 1 h. Lines indicate the mean of *n* = 2 or 3 biological replicates. US: unstimulated. One‐way ANOVA with Tukey's multiple comparison test was performed. Statistical differences are indicated as *p < 0.05. Due to the lower number of repeats, IL10 was excluded from the statistical analysis.

Due to the close proximity of p.K709N to the STAT3 tyrosine 705 (Y705) phosphorylation site and a previously reported phosphorylation defect caused by the heterozygous STAT3 p.K709E variant, we hypothesized that the STAT3 variant p.K709N would also impair STAT3 phosphorylation [24]. Yet analyses by flow cytometry showed intact STAT3 Y705 phosphorylation in patient cells upon stimulation with the cytokines IL6, IL10 and IL21, reaching levels comparable to healthy controls after IL6 stimulation and showing only a trend toward reduced pSTAT3 mean fluorescence intensities (MFI) after stimulation with IL10 and IL21 (Figure 2A; Figure S1). Although STAT3 phosphorylation was not assessed across different cell types or time points, the consistent and pronounced phosphorylation defects observed after IL‐6 stimulation in previous studies using the same experimental setup in patients with the STAT3 p.V637M variant [8] suggest that a similar phosphorylation defect caused by the p.K709N variant is unlikely. Furthermore, the differential impact of STAT3 p.K709N versus p.K709E on phosphorylation is likely due to the latter creating a novel small ubiquitin‐like modifier (SUMO) consensus sequence. The enabled binding of SUMO proteins at K707 thereby likely enhances STAT3 dephosphorylation [25].

### 3.3 STAT3 p.K709N Leads to Reduced STAT3 Target Gene Expression and a Dominant‐Negative Effect on Wild‐Type STAT3

As the reduced number of Th17 cells and the patient's clinical phenotype strongly indicated a STAT3 signaling defect, we tested STAT3 function by assessing the expression of the STAT3 target genes *SOCS3* and *PRDM1*. After stimulation with IL6 or IL10, the expression of both genes was reduced in patient peripheral blood mononuclear cells (PBMCs) compared with control PBMCs, indicating a diminished signaling capacity of the STAT3 p.K709N variant (Figure 2B). To further evaluate the dominant‐negative effect of the mutated STAT3 protein, we transfected the STAT3‐deficient cell line PC‐3 with either wild‐type (wt) STAT3 or equal amounts of STAT3 carrying the p.K709N mutation (mut STAT3) and wt STAT3 to model the heterozygous genotype of the patient. While stimulation with IL6 resulted in an increased expression of the STAT3 target gene *SOCS3* in wt STAT3 only cells, cells transfected with equal amounts of wt and mut STAT3 showed no increase in *SOCS3* expression, suggesting a dominant negative effect for STAT3 p.K709N (Figure 2C). PC‐3 cells expressing the mutated STAT3 p.K709N only showed no upregulation of *SOCS3* upon IL‐6 stimulation (Figure S2).

### 3.4 In Silico Analyses of STAT3 Crystal Structures Suggest an Impaired STAT3 Dimer Formation by STAT3 p.K709N

**FIGURE 3:**
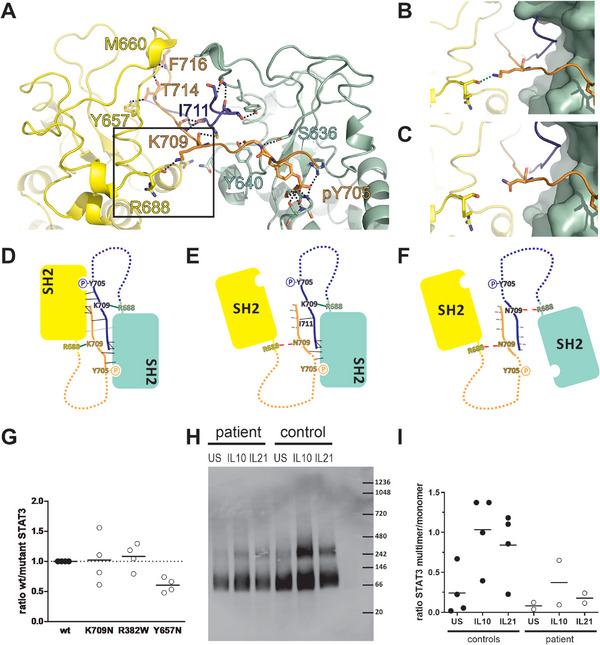
The molecular role of STAT3 Lysine 709 in promoting the homo‐dimer interface. (A) Crystal structure cartoon presentation (PGB entry 1BG1) presented as a zoom‐in on the C‐terminal dimer interface of STAT3. The two STAT3 monomers are colored yellow and green, respectively. Individual sidechains of relevance for the dimer are shown with sticks. (B, C) Zoom‐in of panel (A) highlighting the intramolecular pair of R688 and (B) wt K709 or (C) the N709 substitution. (D–F) Simplified scheme of the C‐terminal dimer interface in the presence of (D) wt K709 or (E, F) the N709 substitution together with a (E) wt monomer or (F) another mutated monomer, leading to monomer destabilization and dimer disruption. (G) Pull‐down experiments using HEK293T cells co‐transfected with plasmids containing myc‐tagged wt STAT3 and either wt‐ or mutant flag‐tagged STAT3 were performed. STAT3 wt‐to‐mutant ratios determined by western blot quantification of the flag‐ and myc‐tagged proteins normalized to WT:mutant ratio of the input are indicated (n = 4 biological replicates). STAT3 p.R382W and p.Y657N plasmids were used as controls for unaffected and impaired dimerization, respectively. (H, I) Native Page analysis of patient or healthy control PBMCs stimulated with either IL10 or IL21 for 20 min was performed. Data are shown as a representative blot of *n* = 2 biological replicates of 2 healthy control and 1 patient sample each (H) and as the calculated multimer/monomer ratio for the patient and the controls (I). Lines indicate the mean of *n* = 2 or 4 biological replicates.

To identify the defective step in the STAT3 signaling cascade resulting from the p.K709N variant, we evaluated STAT3 crystal structures and analyzed the relevance of K709 in STAT3 homo‐dimer formation. In wt STAT3 protein, the C‐terminal tail (CTT), which comprises amino acids L706 to P715, is stabilized by an interaction mediated by a hydrogen bond from the amino group in the amino acid side chain of K709 to the carbonyl oxygen in the main chain of R688 (Figure 3A,B). We suggest that the interaction of K709 with R688 consequently leads to compaction of the STAT3 monomer, facilitating proper positioning of phosphorylated Y705 for its trans‐interaction with the SH2 domain of the reciprocal STAT3 monomer during dimer formation (Figure 3D). Substitution of K709 with the patient's variant N709 in the crystal structure model disrupted the interaction with R688 (Figure 3C–F). The interaction of K709 with R688 was impaired due to a more than two‐fold larger distance to the potential hydrogen bond donor within the N709 side chain compared with the K709 sidechain amino group (Figure 3C–F). Further, N here is a less efficient H‐bond donor given by the longer distance (shorter sidechain). More relevant, based on pKa values, at physiological pH, the K sidechain (pKa ∼10.5–11.0, full cationic) forms a much more efficient salt bridge with the carbonyl of R688 (pKa ∼2.0, full anionic) than N (not protonated, no ion) [26]. Thus, we assume the K‐to‐N substitution causes structural changes to the CTT, resulting in less compacted, destabilized monomers and consequently diminished dimer formation of STAT3 (Figure 3E,F). Our analysis of high‐resolution 3D structures is congruent with previously published data that dimerization of STAT3 molecules depends on interactions of phosphorylated Y705 with the reciprocal SH2 domain. These interactions are supported by *trans* backbone interactions of residues 702–716 between the two interacting monomers and contacts of the C‐terminal tail (CTT) with its own SH2 domain [27]. The importance of the CTT for STAT3 structural integrity and function is also underlined by activating STAT3 mutations in the SH2 domain, which have been shown to stabilize the interaction of the CTT with the SH2 domain, leading to a gain of STAT3 function [27]. Taken together, our in silico analysis suggested an effect of the patient's STAT3 p.K709N variant on dimer formation.

### 3.5 STAT3 p.K709N Likely Impairs STAT3 Dimer Formation and Consequently DNA Binding

To validate the hypothesis from the in silico model indicating an impaired STAT3 dimer formation by the N709‐STAT3 variant, we used an overexpression model in HEK293T cells. As HEK293T cells express low levels of endogenous STAT3, cells were co‐transfected with both a myc‐tagged wild‐type STAT3 plasmid and either a flag‐tagged wild‐type or p.K709N variant STAT3 plasmid. As controls, plasmids containing the STAT3 variants p.Y657N or p.R382W were used. STAT3 p.Y657N had been reported previously to impair STAT3 dimerization, while STAT3 p.R382W showed no dimerization defect (15). STAT3 complexes were isolated from cells by immunoprecipitation using anti‐flag antibodies, and dimers were detected using anti‐myc antibodies. Results using p.K709N variant plasmids varied between the experiments, showing neither a clearly defective nor completely functional dimer formation (Figure 3G). Therefore, we conducted a native page analysis of STAT3 complexes and analyzed PBMCs from the patient after stimulation with IL‐10 and IL‐21. In comparison to healthy subjects, the cells from the patient showed a reduced amount of STAT3 complexes and a reduced ratio of STAT3 multimers to monomers, supporting a defective dimer formation due to the STAT3 p.K709N variant (Figure 3H,I). The molecular weight of the detected STAT3 protein bands indicated that, in addition to monomers (∼80 kDa), rather complexes of four STAT3 monomers than dimers were detected. This is in accordance with previous reports indicating that STAT3 may organize into tetramers upon binding to DNA (28).

Consistent with reduced STAT3 complex formation, STAT3 DNA binding in the patient's PBMCs was diminished after IL‐6 and IL‐10 stimulation compared with healthy controls (Figure S2). This finding aligns with our observation of decreased expression of STAT3 target genes (Figure 2B).

### 3.6 Data Limitations and Perspectives

In this study, all experiments, except for the experiments using STAT3‐deficient cells and the pull‐down assays, were performed using PBMCs from a single patient, while various healthy control individuals were included. Due to variability between experiments and limitations in patient sample availability, observed differences between healthy controls and the patient were not statistically significant. Identification of additional individuals carrying the STAT3 p.K709N variant will enable further experiments to validate the observed functional effects on STAT3 signaling.

## 4 Conclusion

Taken together, our clinical and functional analysis showed that the identified heterozygous STAT3 p.K709N variant caused STAT3‐HIES in the described patient. Our report emphasizes the pivotal role of STAT3 K709 in CTT stabilization and presentation of phosphorylated Y705 to enable STAT3 dimer formation. An interdisciplinary approach is suggested when evaluating genetic variants, including a multidisciplinary team consisting of physicians, geneticists, structural biologists, and molecular biologists.

## Author Contributions

Beate Hagl, Benedikt D. Spielberger, Betina Neumann, Simon J. Pelham, Dharmendra Pandey, Andreas Schlundt, Camille Barro, Anica Lechner, and Christine Wolf performed research and analyzed data. Michael Sattler, Elissa K. Deenick, Stuart G. Tangye, Simon Rothenfusser, and Ellen D. Renner analyzed data. Beate Hagl, Benedikt D. Spielberger, Simon Rothenfusser, and Ellen D. Renner analyzed clinical data. Beate Hagl, Elissa K. Deenick, Stuart G. Tangye, Simon Rothenfusser, and Ellen D. Renner supervised research. Beate Hagl, Benedikt D. Spielberger, Simon Rothenfusser, and Ellen D. Renner designed the research and were the principal writers of the manuscript. All authors reviewed the manuscript and contributed to writing.

## Ethics Statement

This study was performed in line with the principles of the Declaration of Helsinki. Approval was granted by the local review boards (LMU #381‐13, TUM #429/16 S).

## Consent to Participate

Written informed consent was obtained from all individual participants included in the study.

## Consent to Publish Material from Other Sources

Not applicable.

## Conflicts of Interest

The authors declare no conflicts of interest.

## Clinical Trial Registration

Not applicable.

## Supporting information




**Supplementary Information file 1**: eji70015‐sup‐0001‐SuppMat.docx

## Data Availability

The data that support the findings of this study are available on request from the corresponding author. The data are not publicly available due to privacy or ethical restrictions.
