# Supplementary material for: Signal Transducer and Activator of Transcription 3 (STAT3) Variant p.K709N Causes Hyper‐IgE Syndrome Likely by Impaired STAT3‐Dimer Formation
Source: Eur J Immunol. 2025 Jul 28;55(7):e70015. doi: 10.1002/eji.70015 (PMC12304596; doi:10.1002/eji.70015)
Supplement: Supplementary file 1 — Supplementary Information file 1: eji70015‐sup‐0001‐SuppMat.docx [file EJI-55-e70015-s001.pdf]

Supporting information

Material and Methods

Patient, clinical, immunologic and genetic work-up

To evaluate the clinical significance of a genetic variant in *STAT3* (RefSeq NM_139276.2) we performed functional testing in cells of a female patient with findings of HIES from unrelated parents of German descent. This study was performed in line with the principles of the Declaration of Helsinki. The study was approved by the local review boards (LMU #381-13, TUM #429/16 S). Written informed consent was obtained from all individual participants included in the study.

Complete clinical history of the patient was obtained and medical records were reviewed. The patient was also assessed with the previously described NIH-HIES score (1).

Differential blood count and serum immunoglobulin level were assessed. Lymphocyte subsets were analyzed by flow cytometry (BD FACSCalibur and BD FACSCanto, BD Biosciences, San Jose, CA, USA) and compared to age-matched references or healthy controls as previously described (2).

Sanger sequencing

DNA was isolated from peripheral blood using the innuprep Blood DNA Mini kit (Analytik Jena, Jena, Germany). Polymerase chain reaction of the region of interest was performed using AmpliTaq Gold 360 Master Mix (Thermo Fisher scientific, Waltham, MA, USA). The sanger sequencing chromatograms of amplified DNA were received from Eurofins genomics GmbH (Ebersberg, Germany). Primer sequences are available upon request. Mutations were reported using HGSV nomenclature (3).

Whole exome sequencing

Whole exome sequencing (WES) was performed as follows: Approximately 300 bp DNA fragments were generated from the patient’s genomic DNA, which was isolated

from a EDTA blood sample (Transposase, Illumina). These fragments were then enriched using specific probes targeting human exons (the exome). The NGS-Star (Hamilton) system, along with Illumina DNA Prep with Exome 2.5 Enrichment kits and reagents, was used for this process. Sequencing was subsequently carried out on an Illumina NovaSeq 6000 "Next Generation Sequencing" platform.

The bioinformatics analysis of the sequencing data utilized an in-house pipeline (Whole Exome Version V10.1), which includes the Burrows-Wheeler Aligner (BWA 0.7.15), Genome Analysis ToolKit (GATK 3.6), Variant Effect Predictor (VEP 89, www.ensembl.org), and frequency filters based on public and in-house databases (e.g., ExAC and GnomAD).

Isolation and cultivation of PBMCs

Peripheral blood mononuclear cells (PBMCs) were isolated from venous blood using Biocoll Separating Solution (Biochrom AG, Berlin, Germany). Prior to stimulation PBMCs were cultured in serum-free RPMI medium (Thermo Fisher Scientific, Inc., Waltham, USA) overnight.

Evaluation of STAT3 phosphorylation

STAT3 phosphorylation was assessed as previously described (4). In brief: After 20 min. stimulation with 20 ng/ml IL6 or IL10 (both Biochrom) or 10 ng/ml IL21 (R&D Systems, Minneapolis, MN, USA) tyrosine phosphorylation of STAT3 was assessed in patient and control PBMCs by flow cytometry using Alexa Fluor 647 Mouse Anti-Stat3 (pY705) (4/P-STAT3) antibody and BD Phosflow reagents per the manufacturer’s instructions (BD Biosciences) and analysed by flow cytometry (BD FACSCalibur and BD LSR Fortessa, both BD Biosciencies). Data analysis was performed with FlowJo Version 10 (FlowJo LLC, Ashland, OR, USA). Lymphocytes were gated in the forward-scatter and side-scatter (FSC/SSC) plots (Supp. Fig.1a). Histograms indicating the Alexa Flour 647-pSTAT3 signal of the gated lymphocytes were analyzed for mean

fluorescence intensity (MFI). Artificial peaks at 10^0 were excluded from the (MFI) analysis by gating.

Evaluation of STAT3 target gene expression in patient and STAT3-deficient cells

Control and patient PBMCs were stimulated with 20 ng/ml IL6 or IL10 (both Biochrom) for 60 min. RNA was isolated with RNeasy Mini Kit (Qiagen, Hilden, Germany) and cDNA was synthesized using random hexamers (Roche Diagnostics, Penzberg, Germany) and SuperScript II reverse transcriptase (Thermo Fisher Scientific, Inc., Waltham, USA) according to manufacturers' instructions. Quantitative real time-PCR was performed using 2X iTaq SYBR-Green Supermix on a Mastercycler ep realplex2 S (Eppendorf, Hamburg, Germany). Relative target gene expression of *SOCS3 and PRDM1* relative to expression of *TATA-box-binding-protein (TBP)* was performed as described previously (5, 6). Primer sequences are available on request.

The STAT3-deficient cell line PC-3 (ACC 465, DSMZ, Braunschweig, Germany) (7, 8) was cultured in RPMI medium (Thermo Fisher Scientific) supplemented with 10% FBS (Sigma), 1 mM Sodium Pyruvate, 0.1 mM MEM Non-essential amino acid solution and 1% Penicillin/Streptomycin (all Thermo Fisher Scientific) at 37°C in 5% CO2. The STAT3 c.2127G>C variant was introduced into myc- and flag-tagged pcDNA3.1-hSTAT3 plasmids using the QuikChange Lightning Site-Directed Mutagenesis Kit (Agilent, Santa Clara, CA, USA) according to manufacturer's instructions. PC-3 cells were transfected either with wildtype STAT3 plasmid, with equal amounts of wildtype and mutated STAT3 plasmid or with mutated STAT3 plasmid. After 30 hours, cells were stimulated with 20 ng/ml IL-6 for 60 minutes (Active Bioscience, Hamburg, Germany). RNA was extracted using the RNeasy Plus Mini kit (Qiagen) and cDNA was synthesized using the Superscript II Reverse Transcriptase (Thermo Fisher Scientific) according to manufacturer's instructions. Quantitative real time-PCR was performed using PowerUp SYBR Green Master Mix on the QuantStudio 3 Real-Time PCR system

(both Thermo Fisher Scientific). Relative expression of *STAT3* and the STAT3 target gene *SOCS3* was calculated as described previously (6), normalized to the housekeeping genes *β-actin* and *TBP*. Primer sequences are available on request.

Evaluation of STAT3 dimerization

STAT3 pull-down assays were performed as described previously (9). HEK-293T cells were co-transfected with myc-tagged wildtype STAT3 plasmid and either flag-tagged wildtype or mutated STAT3 plasmid. After 48 hours, cells were harvested, rested for 2 hours, and then stimulated with 10 ng/ml IL6 (PeproTech) for 30 minutes. Successful transfection was assessed via flow cytometry and western blotting of whole cell lysates using anti-myc and anti-flag antibodies to ensure comparable amounts of myc- and flag-tagged proteins were present in transfected cells. Cell lysates were immunoprecipitated with anti-flag M2 magnetic beads (Sigma-Aldrich, M8823). Washes were performed in TBS, and captured proteins were eluted using 0.1 M Glycine HCl, pH 3.0. The presence of myc- or flag-tagged WT or flag-tagged mutant STAT3 was assessed by Western blotting on the input and immunoprecipitated output using antibodies against myc (Sigma-Aldrich, clone C3956), flag (Sigma-Aldrich, clone M2) and GAPDH (Santa Cruz, clone 6C5).

Blue-Native PAGE was performed following a previously published protocol (10) with slight modifications using patient and healthy control PBMCs after 20 minutes of stimulation with 20 ng/ml IL10 (Biochrom) or 10 ng/ml IL21 (R&D systems). Briefly, 50 µg of total protein were separated on 4-16% Bis-Tris polyacrylamide gel (Invitrogen) using cathode buffer (50 mM tricine, 7.5 mM Imidazole, 0.02% Coomassie blue-G250, pH 7.0) and anode buffer (25 mM Imidazole pH 7.0) with a constant current of 15 mA. Proteins separated on polyacrylamide gel were electroblotted on PVDF membrane using wet-transfer buffer (25 mM Tris, 192 mM glycine, 20% methanol, pH 8.3) at constant current of 300 mA for 90 minutes. Total STAT3 was detected with an anti-

Stat3 (79D7) antibody (Cell Signaling Technology Inc., MA, USA) and HRP-conjugated anti-rabbit secondary antibody (Bio-Rad Laboratories Inc.). Chemiluminescent protein bands were imaged using ChemiDoc™ imaging system (Bio-Rad Laboratories Inc.). NativeMark™ unstained protein standard (Thermo Fischer Scientific) was used as a size standard. Band intensities were quantified using Image lab 6.1 software (Bio-Rad Laboratories Inc.). For normalization monomer to multimer ratios were calculated using the intensity of the monomer bands as internal controls.

Analysis of STAT3 DNA binding capacity

PBMCs were stimulated with 20 ng/ml IL6 or IL10 (both Biochrom) or 10 ng/ml IL21 (R&D Systems) for 20 min and 60 min, respectively. Nuclear extracts of control and patient PBMCs were isolated using the Nuclear Extract Kit (Active Motif, La Hulpe, Belgium) according to the manufacturer's instructions. Protein concentrations were assessed with Bio-Rad Protein Assay Kit II (Bio-Rad Laboratories, Hercules, CA, USA). STAT3 DNA binding capacity was examined using the TransAM STAT3 Transcription Factor Assay Kit (Active Motif) using 2 or 1 µg of nuclear protein per well according to manufacturer's instructions.

***In silico* analyses**

The SIFT, PolyPhen-2, Mutation Assessor, PROVEAN, CADD, Condel, FATHMM, CADD, and AlphaMissense algorithms were used to predict if the detected mutation is disease-causing and the gnomAD and ClinVar variants were searched (11-18). For the *in silico*-based analysis of NP_644805.1:p.(K709N) effects on the propensity of STAT3 to form dimers, we inspected published crystal structures of STAT3 dimers bound to DNA. From the available human and murine complex structures deposited in the Protein Data Bank (19) we selected entry 1BG1 (20) yielding the highest resolution (2.25 Å) and taking into account a 100-% sequence identity between murine and human STAT3 within the resolved region of residues 127-715 (identical in all available

entries). The crystal structure was used for *in silico* substitution of amino acid 709, identification of interaction networks and generation of figures using PyMOL (Schrödinger, LLC).

References

1. Grimbacher B, Schaffer AA, Holland SM, Davis J, Gallin JI, Malech HL, et al. Genetic linkage of hyper-IgE syndrome to chromosome 4. Am J Hum Genet. 1999;65(3):735-44.
2. Hagl B, Heinz V, Schlesinger A, Spielberger BD, Sawalle-Belohradsky J, Senn-Rauh M, et al. Key findings to expedite the diagnosis of hyper-IgE syndromes in infants and young children. Pediatr Allergy Immunol. 2016;27(2):177-84.
3. den Dunnen JT, Dalgleish R, Maglott DR, Hart RK, Greenblatt MS, McGowan-Jordan J, et al. HGVS Recommendations for the Description of Sequence Variants: 2016 Update. Hum Mutat. 2016;37(6):564-9.
4. Hagl B, Spielberger BD, Thoene S, Bonnal S, Mertes C, Winter C, et al. Somatic alterations compromised molecular diagnosis of DOCK8 hyper-IgE syndrome caused by a novel intronic splice site mutation. Scientific reports. 2018;8(1):16719.
5. Pfaffl MW. A new mathematical model for relative quantification in real-time RT-PCR. Nucleic Acids Res. 2001;29(9):e45.
6. Livak KJ, Schmittgen TD. Analysis of relative gene expression data using real-time quantitative PCR and the 2(-Delta Delta C(T)) Method. Methods. 2001;25(4):402-8.
7. Spiotto MT, Chung TD. STAT3 mediates IL-6-induced growth inhibition in the human prostate cancer cell line LNCaP. Prostate. 2000;42(2):88-98.
8. Clark J, Edwards S, Feber A, Flohr P, John M, Giddings I, et al. Genome-wide screening for complete genetic loss in prostate cancer by comparative hybridization onto cDNA microarrays. Oncogene. 2003;22(8):1247-52.
9. Pelham SJ, Lenthall HC, Deenick EK, Tangye SG. Elucidating the effects of disease-causing mutations on STAT3 function in autosomal-dominant hyper-IgE syndrome. J Allergy Clin Immunol. 2016;138(4):1210-3 e5.
10. Wittig I, Braun HP, Schagger H. Blue native PAGE. Nat Protoc. 2006;1(1):418-28.
11. Sim NL, Kumar P, Hu J, Henikoff S, Schneider G, Ng PC. SIFT web server: predicting effects of amino acid substitutions on proteins. Nucleic Acids Res. 2012;40(Web Server issue):W452-7.
12. Adzhubei IA, Schmidt S, Peshkin L, Ramensky VE, Gerasimova A, Bork P, et al. A method and server for predicting damaging missense mutations. Nat Methods. 2010;7(4):248-9.
13. Reva B, Antipin Y, Sander C. Predicting the functional impact of protein mutations: application to cancer genomics. Nucleic Acids Res. 2011;39(17):e118.
14. Choi Y, Sims GE, Murphy S, Miller JR, Chan AP. Predicting the functional effect of amino acid substitutions and indels. PLoS One. 2012;7(10):e46688.
15. Schubach M, Maass T, Nazaretyan L, Roner S, Kircher M. CADD v1.7: using protein language models, regulatory CNNs and other nucleotide-level scores to improve genome-wide variant predictions. Nucleic Acids Res. 2024;52(D1):D1143-D54.
16. Gonzalez-Perez A, Lopez-Bigas N. Improving the assessment of the outcome of nonsynonymous SNVs with a consensus deleteriousness score, Condel. Am J Hum Genet. 2011;88(4):440-9.

17. Shihab HA, Gough J, Cooper DN, Stenson PD, Barker GL, Edwards KJ, et al. Predicting the functional, molecular, and phenotypic consequences of amino acid substitutions using hidden Markov models. Hum Mutat. 2013;34(1):57-65.
18. Cheng J, Novati G, Pan J, Bycroft C, Zemgulyte A, Applebaum T, et al. Accurate proteome-wide missense variant effect prediction with AlphaMissense. Science. 2023;381(6664):eadg7492.
19. Berman HM, Westbrook J, Feng Z, Gilliland G, Bhat TN, Weissig H, et al. The Protein Data Bank. Nucleic Acids Res. 2000;28(1):235-42.
20. Becker S, Groner B, Muller CW. Three-dimensional structure of the Stat3beta homodimer bound to DNA. Nature. 1998;394(6689):145-51.

Supplementary Table

Prediction tool	Reference	Score	Predicted effect
SIFT	[1]	0.29	tolerated
Polyphen-2	[2]	0.000	benign
Mutation Assessor	[3]	1.525	low functional impact
PROVEAN	[4]	-1.58	tolerated
CADD modeling	[5]	22.1	deleterious
Condel	[6]	0.562	deleterious
FATHMM	[7]	-4.02	damaging
AlphaMissense	[8]	0.882	likely pathogenic

[1] Sim NL, Kumar P, Hu J, Henikoff S, Schneider G, Ng PC. SIFT web server: predicting effects of amino acid substitutions on proteins. Nucleic Acids Res. 2012 Jul;40(Web Server issue):W452-7.

[2] Adzhubei IA, Schmidt S, Peshkin L, Ramensky VE, Gerasimova A, Bork P, Kondrashov AS, Sunyaev SR. A method and server for predicting damaging missense mutations. Nat Methods. 2010 Apr;7(4):248-9.

[3] Reva B, Antipin Y, Sander C. Predicting the functional impact of protein mutations: application to cancer genomics. Nucleic Acids Res. 2011 Sep 1;39(17):e118.

[4] Choi Y, Sims GE, Murphy S, Miller JR, Chan AP. Predicting the functional effect of amino acid substitutions and indels. PLoS One. 2012;7(10):e46688.

[5] Schubach M, Maass T, Nazaretyan L, Röner S, Kircher M. CADD v1.7: using protein language models, regulatory CNNs and other nucleotide-level scores to improve genome-wide variant predictions. Nucleic Acids Res. 2024 Jan 5;52(D1):D1143-D1154.

[6] González-Pérez A, López-Bigas N. Improving the assessment of the outcome of nonsynonymous SNVs with a consensus deleteriousness score, Condel. Am J Hum Genet. 2011 Apr 8;88(4):440-9.

[7] Shihab HA, Gough J, Cooper DN, Stenson PD, Barker GL, Edwards KJ, Day IN, Gaunt TR. Predicting the functional, molecular, and phenotypic consequences of amino acid substitutions using hidden Markov models. Hum Mutat. 2013 Jan;34(1):57-65.

[8] Cheng J, Novati G, Pan J, Bycroft C, Žemgulytė A, Applebaum T, Pritzel A, Wong LH, Zielinski M, Sargeant T, Schneider RG, Senior AW, Jumper J, Hassabis D, Kohli P, Avsec Ž. Accurate proteome-wide missense variant effect prediction with AlphaMissense. Science. 2023 Sep 22;381(6664):eadg7492.

Supplementary Table: Variable outcomes in *in silico* variant prediction.

Four of eight variant effect prediction tools classified the STAT3 p.K709N as deleterious or damaging, while four tools predicted a benign effect or low impact of the variant.

Supplementary Figure 1

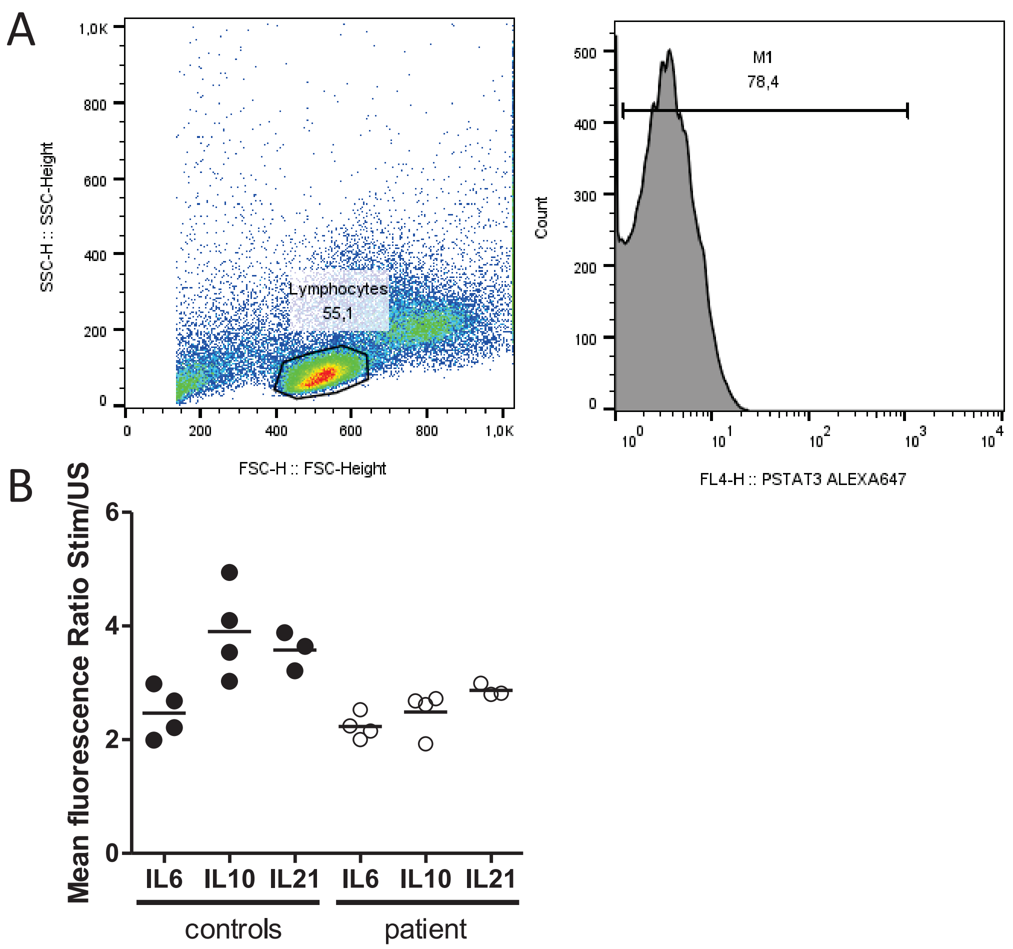

Supplementary Figure 1: Gating strategy and mean fluorescence intensity ratio of pSTAT3 flow cytometric analysis.

(A) Lymphocytes were gated in the forward-scatter and side-scatter (FSC/SSC) plots. Histograms indicating the Alexa Flour 647-pSTAT3 signal of the gated lymphocytes were analyzed for mean fluorescence intensity (MFI). Artificial peaks at 10^0 were excluded from the (MFI) analysis by gating. (B) Lines indicate the mean of n=3 or 4 biological replicates. Kruskal-Wallis test with Dunn's multiple comparisons test did not indicate significant differences between controls and patient.

Supplementary Figure 2

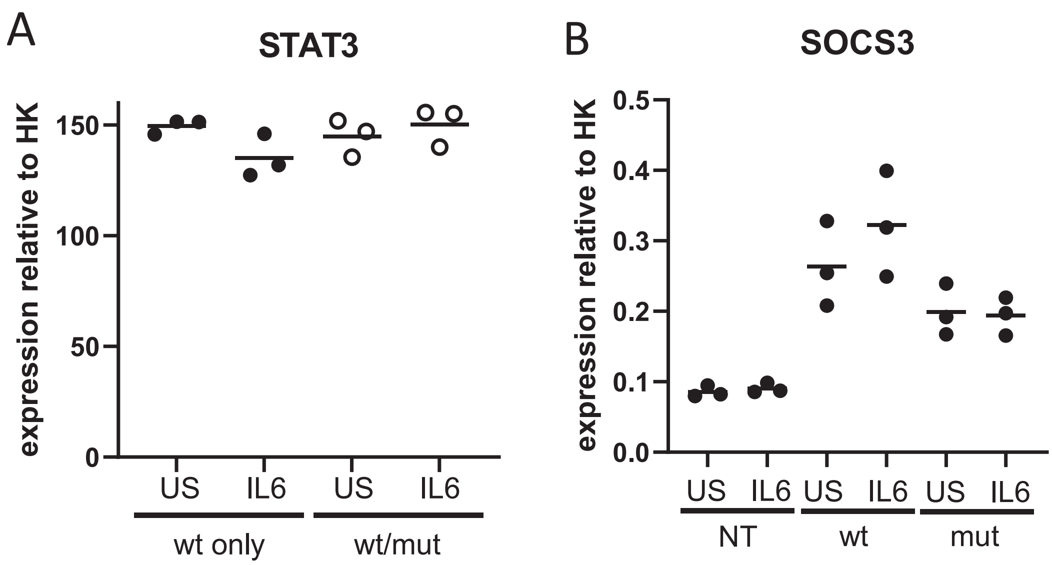

Supplementary Figure 2: Expression of *STAT3* and *SOCS3* mRNA after transfection with wt and p.K709N mut STAT3 plasmids.

(A) Expression of *STAT3* in PC-3 cells transfected with wt or equal amounts of wt and p.K709N mut STAT3 plasmids. (B) Expression of the STAT3 target gene *SOCS3* in not transfected (NT) PC-3 cells and PC-3 cells transfected with wt or p.K709N mut STAT3 plasmids. Gene expression was normalized to the house keeping genes (HK) *TBP* and *b-Actin.* 30 hours after transfection cells were stimulated with IL6 for 1 hour. Lines indicate the mean of n=3 biological replicates. US: unstimulated.

Supplementary Figure 3

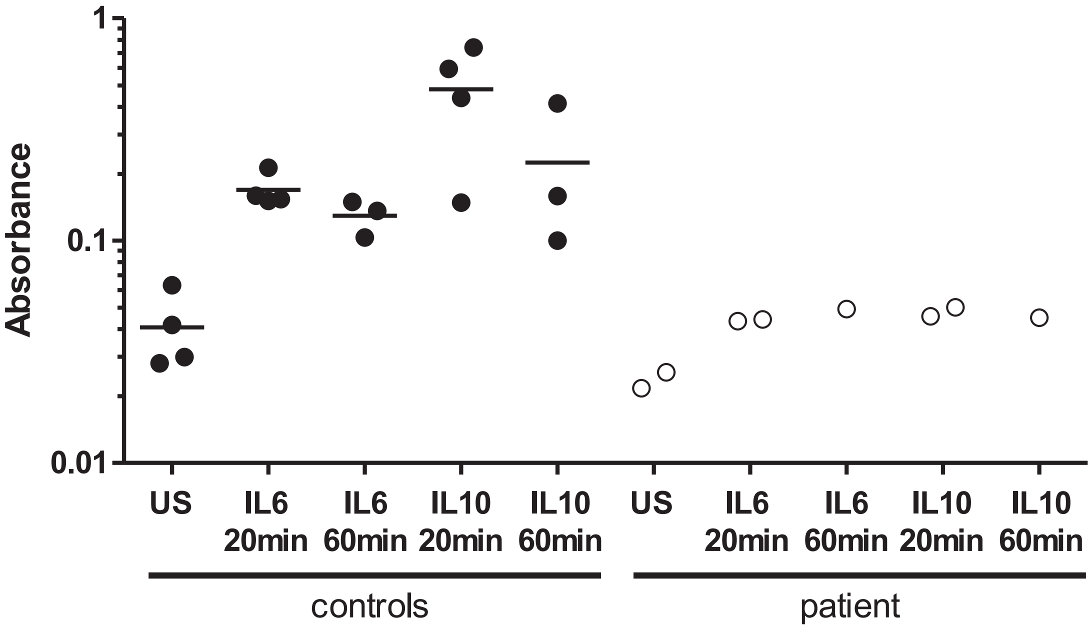

Supplementary Figure 3: DNA binding capacity in patient PBMCs.

STAT3 DNA binding assays of PBMCs stimulated with IL6 and IL10 for 20 and 60 min were performed to analyze DNA binding capacity in patient PBMCs (n=2 or n=1 biological replicates) compared to four healthy controls (n=3 or 4 biological replicates). Lines indicate the mean of independent experiments.
